# Effectiveness of Mandatory and Incentive-Based Routine Childhood Immunization Programs in Europe: A Systematic Review of the Literature

**DOI:** 10.3390/vaccines9101173

**Published:** 2021-10-13

**Authors:** Lidia Kuznetsova, Giorgio Cortassa, Antoni Trilla

**Affiliations:** 1 Barcelona Institute for Global Health, Hospital Clínic, University of Barcelona, 08036 Barcelona, Spain; ATRILLA@clinic.cat; 2 Emergency Department, Hospital Santa Corona, 17027 Pietra Ligure, Italy; cortassa@hotmail.com

**Keywords:** child immunization, mandatory vaccination, vaccine-preventable diseases, vaccination coverage, incidence, COVID-19, Europe

## Abstract

There is a lack of comprehensive and systematic data and evidence regarding the effectiveness of mandatory and incentive-based vaccination schemes. The results of such programs in some countries have not been adequately studied. A number of countries have recently introduced tightening vaccination measures, and it is important to analyze and assess the results of these programs. The unprecedented situation due to the COVID-19 pandemic and mass vaccination made the topic of the effectiveness of vaccination policies and mandates even more relevant. The aim of the study is to assess childhood vaccination programs implemented in selected countries. The study focuses on initiatives implemented in the European Region of the World Health Organization (WHO). A total of 466 full-text articles were assessed for eligibility, and 26 articles on seven countries were included in the synthesis. Additionally, we obtained and performed an analysis of data on the impact of COVID-19 on vaccine coverage and incidence of vaccine-preventable diseases, and the implementation of vaccine mandates in the selected countries. The evidence collected and analyzed in this review allowed us to conclude that the introduction of children routine vaccination mandates increases vaccine coverage and reduces the incidence of vaccine-preventable diseases when compared to the situation before the introduction of the mandates.

## 1. Introduction

Currently, there are no universally acceptable vaccination policies that have proved the most effective to achieve target vaccination coverage rates. The choice of vaccination policy depends on local conditions and characteristics of the countries or regions where the programs are implemented such as the level of trust in government, historical factors, level of economic development, country size, etc. Some governments, like those in Scandinavian countries, opted for recommended vaccination programs and maintain high vaccination coverage (VC) and low morbidity for vaccine-preventable diseases. On the other hand, struggling to achieve target immunization coverage rates and experiencing outbreaks, some countries, like France, Italy, and Ukraine adopted mandatory vaccination policies. Policy enforcement measures are also tailored according to countries characteristics. The general trend in many countries, including European countries, is tightening immunization policies by introducing mandatory vaccinations or incentives/penalty-based measures. This process has been triggered by vaccine hesitancy, which was announced by the WHO as one of ten threats to global health in 2019 [1].

The introduction of mandates is also being driven by the public opinion in many countries that supports mandatory immunization policies [2]. Vaccine hesitancy provoked recent outbreaks of vaccine-preventable diseases around the world. In 2019, a record number of measles cases was registered in the European Region of WHO [3]. Cases of diphtheria have increased in recent years. The number of antimicrobial resistance genes, as well as the breadth of antibiotic resistance, is substantially greater in the last decade than ever before [4]. These outbreaks revealed the necessity to review current vaccination policies in some countries to respond more effectively.

The unprecedented situation due to the COVID-19 pandemic and mass vaccination made the topic of the effectiveness of vaccination policies and mandates especially relevant. A number of countries introduced COVID-19 vaccine mandates, which caused extraordinary public response [5]. The results of the introduction of these mandates can be analyzed shortly. The COVID-19 pandemic made the question of the effectiveness of vaccine mandates even more relevant, as many countries in the world faced the necessity to achieve adequate COVID-19 vaccine coverage in response to the pandemic. As regards vaccinations according to vaccination schedules, many countries have experienced significant vaccination coverage reduction during the pandemic [6]. At the same time, COVID-19 protective measures, such as social distancing, hygiene, etc., led to a drastic reduction in the incidence of vaccine-preventable diseases [7,8].

Therefore, there is a need to study the effectiveness of routine vaccination programs, and in particular, mandatory and incentive-based measures. It is difficult to underestimate the relevance of effectiveness of vaccination policies and vaccine mandates. The results of the implementation of such policies in a number of European countries in recent years provide new data for more comprehensive analysis.

Most researchers agree that there is a lack of comprehensive and systematic data and evidence regarding the effectiveness of mandatory and incentive-based vaccination schemes. The results of such programs in some countries have not been adequately studied. A number of countries have only recently introduced tightening vaccination measures, and it is important to analyze and assess the results of these programs.

The aim of the study is to assess childhood vaccination programs implemented in selected countries and to understand what policies are the most effective and suitable for different contexts.

## 2. Methods

The review has been performed following the guidance issued by the Preferred Reporting Items for Systematic Reviews and Meta-Analyses (PRISMA) and Cochrane Handbook for Systematic Reviews of Interventions, including its technical supplements [9,10].

The research question of the systematic review is as follows: What is the effect of mandatory and incentive-based routine childhood immunization programs on vaccination coverage and morbidity for vaccine-preventable diseases in children in the selected countries?

### 2.1. Eligibility Criteria

Studies have been included if they compared vaccination coverage, incidence, or seroprevalence of vaccine-preventable diseases before and after the introduction of vaccine mandates or incentive-based measures. Mandates are defined as official requirements, codified in law, for children to be vaccinated. We distinguish positive incentives in the form of rewards for vaccinating and negative incentives in the form of penalties such as detention, exclusion from schools or other social gatherings, or monetary fines. Studies have been included if they analyzed data from countries of the European Region of the WHO and were written in English, French, German, Italian, Russian, Spanish, or Ukrainian. To be eligible, studies should have analyzed data at the national or regional level. Multi-country studies have also been included. The included studies have analyzed data on one or more of the following diseases/vaccines: measles, mumps, rubella, diphtheria, polio, pertussis, tetanus, varicella, hepatitis B (HepB), and Haemophilus influenzae type b (Hib). Published and unpublished studies from 2000 to 2021 were eligible.

### 2.2. Information Sources

The sources of information included databases, manual search in catalogues, contact with the authors of the studies, etc. The search has been performed in MEDLINE (OVID) (Appendix A), Web of Science (Appendix B), PubMed, and a regional database, eLIBRARY.RU. Additionally, we have searched Google Scholar and Google, as sources of gray literature on mandatory/incentive-based vaccination programs in the relevant countries. We have contacted research groups in a number of countries with questions regarding vaccination mandates in respective countries and relevance of their studies to the results of vaccination policies. Researchers have also been contacted for information about unpublished or ongoing studies in order to reduce the risk of publication bias and to identify as much relevant evidence as possible. We have gone through the reference lists of some of the included studies and relevant systematic reviews identified to find more relevant studies and consult information about the search strategies used, to inform the systematic review.

### 2.3. Study Selection, Data Collection, and Analysis

Firstly, the studies were screened by titles, secondly, by abstracts, and thirdly full-text screen was conducted. A second reviewer validated a random sample of 20% of studies from the first and second screenings. Discrepancies were resolved by discussion. Where publications lacked the information for making a decision, the authors were contacted for further details.

Data have been extracted into a piloted and then a standardized form, which included the following categories: country, population, study period, study design, vaccine/disease, intervention, outcomes, and results. Data were extracted independently by two reviewers, with consensus reached by discussion.

Data have been synthesized qualitatively due to heterogeneity in study population, vaccines/diseases analyzed, and study design.

## 3. Results

**Figure 1:**
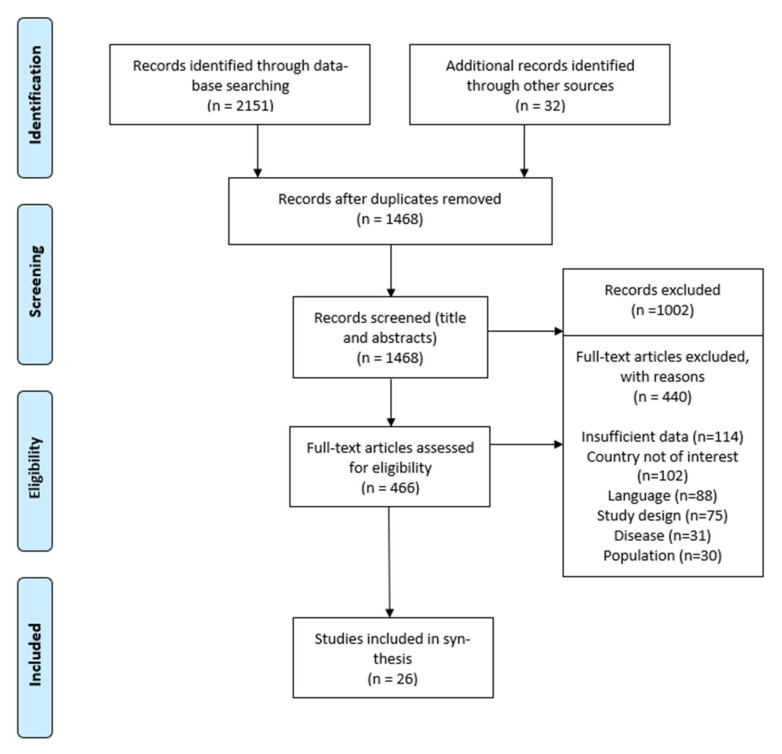
Flow diagram showing the review process.

The search identified 2151 records. After removing duplicates, 1468 titles and abstracts were screened. A total of 466 full-text articles were assessed for eligibility, and 26 studies were included in synthesis (Figure 1). Characteristics of included studies are presented in the Table 1. The review included studies from seven countries: seven studies on Italy, four on Germany, three on France, four on Latvia, three on Serbia, three on Moldova, and two on Ukraine. The data from some countries that fit the search and inclusion criteria were not available, such as Slovakia and Slovenia, that introduced tightening vaccination policies in 2020. The review included registry analysis studies, surveys, and retrospective studies. A total of 19 studies analyzed data on vaccine coverage, 16 on incidence, and 3 on seroprevalence. The included countries introduced vaccination mandates and negative incentives. France, Moldova, Ukraine, and Latvia require vaccination certificate for children to attend educational institutions. Germany, Italy, and Serbia introduced monetary fines for not vaccinating children, in addition to vaccination certificate requirements. Germany and Ukraine introduced mandates just recently, in 2020 and 2019, respectively. Italy, France, and Serbia introduced mandatory vaccination in the last 5 years. Latvia and Moldova introduced such measures over a decade ago. Germany and Ukraine are the countries with different characteristics, but the introduction of vaccination mandates in these countries shows similar effects and trends, including the effect of COVID-19. The general trend during the period covered by the review is tightening children vaccination policies throughout the WHO European Region.

### 3.1. France

**Figure 2:**
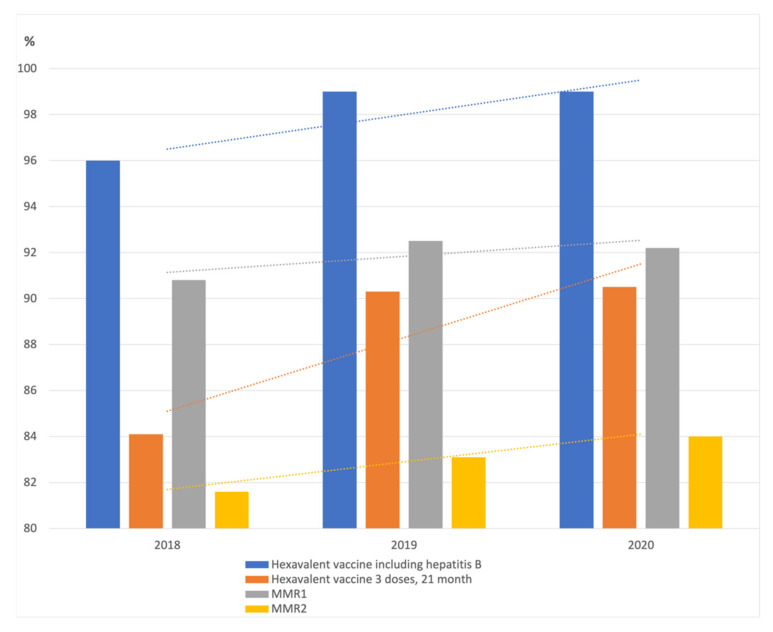
Vaccination coverage for hexavalent and measles, mumps and rubella vaccines in France, 2018–2020. Data: Santé publique France—SNDS (DCIR).

In December 2017, French parliamentarians passed a law extending the vaccination mandates for children from 3 (diphtheria, tetanus, and poliomyelitis) to the 11 vaccinations included in the routine immunization schedule of children under 2 years old. Children born from 1 January 2018 onwards are required to receive three doses of a hexavalent vaccine which includes diphtheria, tetanus, poliomyelitis, pertussis, Haemophilus influenza b, and hepatitis B antigens at ages 2 and 4 months, with a booster dose at 11 months; three doses of the vaccine against invasive pneumococcal diseases with the same schedule; two doses of a vaccine against meningococcal C diseases at ages 5 and 12 months; two doses of MMR vaccine at age 12 and 16–18 months [33]. The included studies examined the effect of this law on the vaccine coverage. The two studies from France, a survey and a registry analysis, come to the same conclusion, the positive effect of the law. Both studies indicate a significant increase in HepB vaccine coverage, approximately by 6% after the introduction of the law. Both studies also mention an increase of 3–4% in MMR vaccine coverage between 2017 and 2019 [11,12]. Cohen et al. argue that in 2017–2019 VC for pertussis and Hib components, was high and stable (≥94%). Figure 2 and Table 2 show the impact of the mandatory vaccination law in France.

The authors conclude that the increase in VC was associated with a regain of confidence of the population in vaccination, as shown by the increased proportion of mothers in favor to vaccination and thinking to be rather well/perfectly well informed on vaccination and the decreased proportion of mothers against mandatory vaccination between 2017 and 2019. The authors add that the extension of mandatory vaccination has been well accepted by health care professionals and the general population. Lévy-Bruhl D et al. argue that the measles resurgence which started at the end of 2017 may have contributed to the increase in MMR VC. The authors indicate especially remarkable increasing trend seen for vaccination coverage of children too old to have been concerned by the mandates. The increasing trend of vaccine coverage observed since 2018 has been weakened as a consequence of the pandemic in France [13]. The authors argue that to date, the new law on mandatory vaccination has no negative effect on vaccine coverage for vaccinations not yet concerned by the mandates or which remain recommended.

### 3.2. Germany

**Figure 3:**
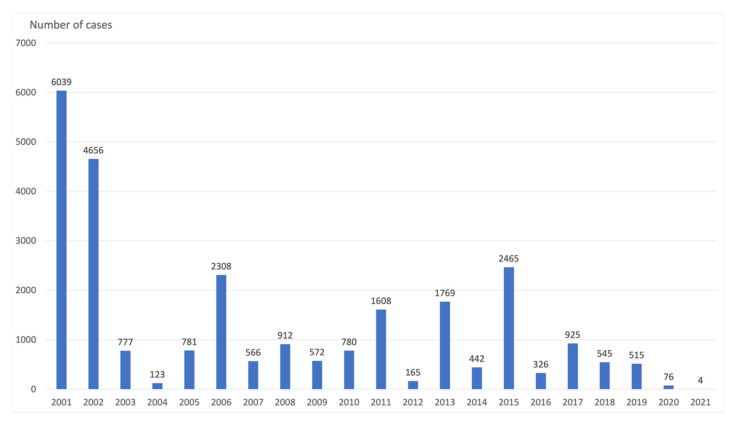
Number of cases of measles in Germany, 2001–2021. For 2021: data as of July 1st, 2021. Source: Robert Koch-Institute.

The law adopted to protect against measles and to strengthen vaccination prevention of 10 February 2020 is a German federal law that has been in force since 1 March 2020. With Art. 1, the Infection Protection Act was changed and expanded to include provisions on the mandatory vaccination for protection of certain categories of the population against measles as a benefit of the statutory health insurance. It stipulates that all children from the age of one must provide evidence of the measles vaccination recommended by the Standing Vaccination Commission when entering kindergarten. Students starting their first year of school also must prove measles vaccination [34]. Three registry analysis studies and one survey examine incidence and vaccine coverage before and after the introduction of the regulation. Endemic rubella has been eliminated in Germany since 2020. The number of reported rubella cases was at a low level in 2018 and 2019 (n = 58 cases). In 2020, 18 rubella cases were reported to the Robert Koch Institute (RKI). In 2020, the number of measles cases in Germany has decreased by 85.5% compared to previous years since 2016. This is the lowest annual number of cases that has been reported to the RKI since the introduction of mandatory reporting in 2001. A backlog of cases at the health authorities was unlikely because at that time the measles protection law that came into force at the beginning of March meant that attention was high and acute measles cases belonged to the high-priority reporting categories in the federal states. Furthermore, the influence of the Measles Protection Act on the decline in the number of cases from March 2020 onwards was assessed as low overall [14]. Neugebauer et al. argue that the punitive measures under the Measles Protection Act seem to have had a positive effect on the participants in their study. Since the measles vaccination rate in children from 3 to 17 years of age is already close to the necessary 95%, no opponent of vaccination may have to be persuaded by sanctions. According to the authors, the aim is to reach the parents who have not yet had their children vaccinated for other reasons. The responses from vaccine skeptics in this study show that they are willing to take on a lot before vaccinating their children. The Measles Protection Act created a legally binding regulation that adults should be vaccinated against measles and thus the immunity can be significantly increased. The willingness of parents to vaccinate their children, on the other hand, is significantly lower. The sanctions in the Measles Protection Act also seem to be able to improve little in this regard [15]. According to RKI, there has been a 7-fold decrease in measles cases in 2020 compared to 2019, a 2.5-fold decrease in rubella cases, a 3-fold decrease in pertussis cases, a 2-fold decrease in Hib diseases, and a 2-fold decrease in varicella [7]. This trend continued in 2021 [16]. Table 3 shows the incidence of measles, mumps, and rubella in Germany in 2019–2021. Figure 3 shows the incidence of measles in Germany in 2001–2021.

According to Matysiak-Klose D. et al., the dramatic decrease in the incidence of vaccine-preventable diseases in Germany with an especially sharp decrease in measles cases can be mainly attributed to COVID-19 preventive measures [14].

### 3.3. Italy

**Figure 4:**
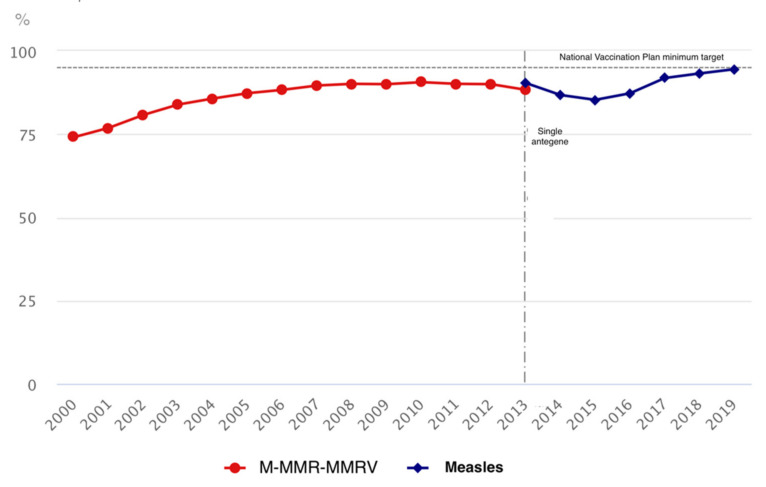
Measles vaccine coverage at 24 months of age, Italy, 2000–2019. Source: EpiCentro—Italian National Institute of Health.

In July 2017, in response to the alarming drop in VC, the Italian Ministry of Health approved the law N 119, which has extended the number of mandatory vaccinations for school attendance from four to ten. In particular, vaccination against poliomyelitis, diphtheria, tetanus, pertussis, hepatitis B, Hib disease, measles, mumps, rubella, and varicella became mandatory for kindergarten attendance. For children and adolescents attending primary and secondary schools (6–16 years), monetary fines for families of unvaccinated children were imposed [35]. The included studies analyze the effect of this law on the national level (four studies) and regional level (three studies). The review included five registry analysis studies, one retrospective, and one survey. The included articles examined the impact of vaccines mandates on vaccine coverage, incidence, and seroprevalence. All the studies concluded that the effect of the law on these indicators was positive. MMR coverage was 91.6% for the year 2017, showing a 4.3% increase compared with 2016 (87.3%). Figure 4 shows measles vaccine coverage at 24 months of age. An increase in other vaccines’ coverage from 1% to 2% was reported [22].

The increase has been consistent—although at different rates—in all regions and is highest for MMR vaccine, as compared to other vaccines. The increasing trend in vaccine coverage has continued in 2018. Table 4 shows vaccination coverage and incidence for the diseases covered in this section in Italy in 2016–2019.

Vaccination coverage rates for full vaccination cycle of MMRV in the Palermo Local Health Unit, among 6-year-old children, showed an increase from 61% to 89.7% (+28.7%) from 2016 to 2018 [18]. However, Gori et al. argue that a potential decrease in the coverage rates may be observed as a result of an attenuation of the positive effects of mandates over time. Thus, it is important to combine these measures with information campaigns and political initiatives at different levels, both national and regional [20]. Furthermore, VC for measles-containing vaccine increased by 5.7% in 2018 compared to 2016 with regard to the 1st dose (87.3% in 2016 vs. 93.0% in 2018) and by 6.6% with regard to the 2nd dose (82.2% in 2016 vs. 88.9% in 2018) [17]. The authors point out that compared to previous years, a dramatic increase in the incidence of measles was observed in 2017, when measles incidence reached 88.4 cases per million population. In 2018, measles incidence decreased to 43.3 cases per million population. The study conducted by Gianfedi et al. indicated an important reduction in missed vaccination after the introduction of the mandatory vaccination law for both polio and measles with the consequent increase of VC [19]. The reduction was evident especially for measles (−4.1%). The authors argue that this decrease is likely due to the introduction of the law increasing the number of mandatory vaccinations. Furthermore, Signorelli et al. examined the effect of suspension in 2007 in Veneto Region with a regional law national-level mandatory immunization against polio, hepatitis B, tetanus, and diphtheria for children. The authors concluded that although after the implementation of the regional law in 2007 encouraging results were initially reported with no significant drop in coverage rates in Veneto, as compared to previous years, long-term data for Veneto Region show greater decrease in coverage rates (−5.0% for polio vaccine between 2006 and 2016), as compared to figures reported at the national level (−3.2% for polio vaccine in the same study period) and in other neighboring regions. Therefore, the data suggest that the policy of suspending mandatory vaccination to encourage proactive vaccine uptake has not been successful [22].

### 3.4. Latvia

**Figure 5:**
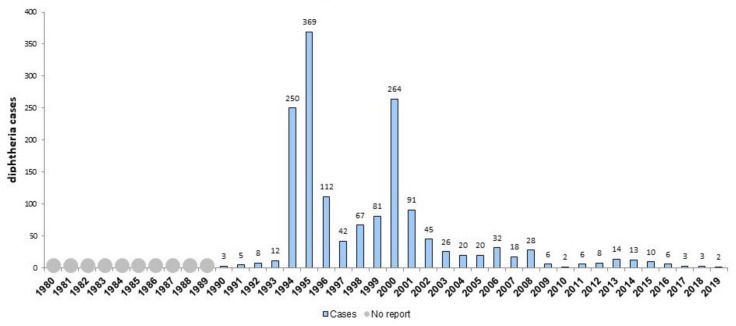
Number of reported diphtheria cases in Latvia, 1980–2019. Source: WHO.

**Figure 6:**
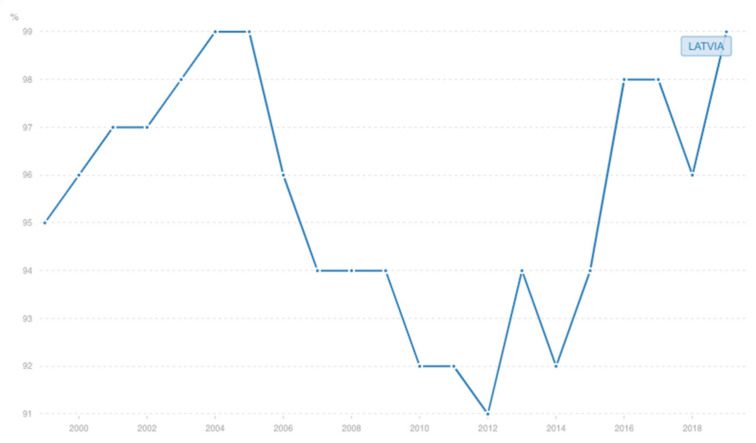
Diphtheria-pertussis-tetanus vaccine coverage (children ages 12–23 months) in Latvia, 2000–2018. Data: WHO.

In 2000 Latvia adopted “Vaccine Regulation” No. 330 which specifies that within the framework of the State Immunization Programme vaccination shall be mandatory for children against 10 vaccine-preventable diseases [36]. Four registry analysis studies on Latvia report vaccine coverage and incidence. A wave of increasing incidence of diphtheria was observed in 2000 (11.1/100,000). From 2000 to 2014, childhood vaccination coverage with a third dose of the vaccine ranged from 91% to 98% and with a fifth dose from 92% to 98%. From 2000 to 2014, vaccination coverage for adolescents (sixth dose at 15 years) ranged from 86% to 96%, a decrease in coverage occurred from 96% in 2007 to 86% in 2014. Therefore, diphtheria incidence decreased in Latvia from 111.22/million in 2000 to 2.67/million in 2009 [26]. Diphtheria was kept under control in the next years. Heininger at al. reported an approximately ten-fold decrease in the reported annual pertussis incidence rate between 2000 and 2011, before a dramatic increase in 2012 [24]. According to the WHO report, the coverage for all the vaccines included in this systematic review increased in 2000 and 2001. DTP1 increased by 7%. There was a dramatic increase of incidence of mumps and pertussis in 2000. The incidence continued growing in 2001 and 2002 and decreased in the next years [27]. The data show overall positive effect of the introduced regulation, especially on vaccine coverage. Figure 5 and Figure 6 show the incidence of diphtheria and DTP vaccine coverage in Latvia. Table 5 shows vaccination coverage and number of cases of corresponding diseases in Latvia in 1998–2002.

### 3.5. Moldova

**Figure 7:**
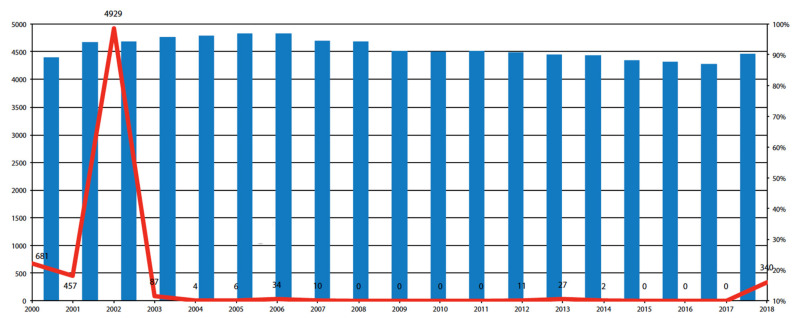
The number of measles cases and coverage of measles immunization for children aged 15 months in the Republic of Moldova, 2000–2018. Note: Columns—the level of the immunization coverage in %; line—number of measle cases. Source: Melnik A. et al. [29].

In 2009, the Parliament of Moldova passed the Law on State Surveillance of Public Health requiring children to receive all vaccines in the national schedule to enroll in kindergartens and schools [37]. Three registry analysis studies on Moldova examined vaccine coverage and incidence. In the period of 2003–2017, the incidence of measles dropped sharply, with a total of 181 cases reported, that is, the average annual incidence was 12 cases. No measles cases were reported in 2008–2011. Melnik et al. report a gradual decrease in the level of coverage with the first dose of MMR in 2003–2017, the lowest rate is observed in 2017—87.1%. Figure 7 shows measles incidence and VC in Moldova in 2000–2018.

In 2009–2012 vaccine coverage was stable at the level of 90%. The authors argue that the use of highly effective modern measles vaccines and the vaccination policy made it possible to achieve in Moldova the status of a country that eliminated measles in 2008–2017 [29]. The incidence of pertussis dropped in 2009–2010 from 47 to 31 cases and increased in the following years. In 2009–2013, pertussis VC was above 90%, and under 90% in the following years. Progressive reduction of the vaccine coverage observed in 2008–2015, from 95.4% to 89.7%, caused an increase in the incidence of pertussis. The data from the WHO surveillance report show no clear effect associated with the introduction of the regulation. The trends in coverage and incidence are uneven: with coverage for some vaccines remaining the same in the year of introduction, some—increasing and some—decreasing [27]. Table 6 shows vaccination coverage and number of cases of corresponding diseases in Moldova in 2007–2011.

### 3.6. Serbia

**Figure 8:**
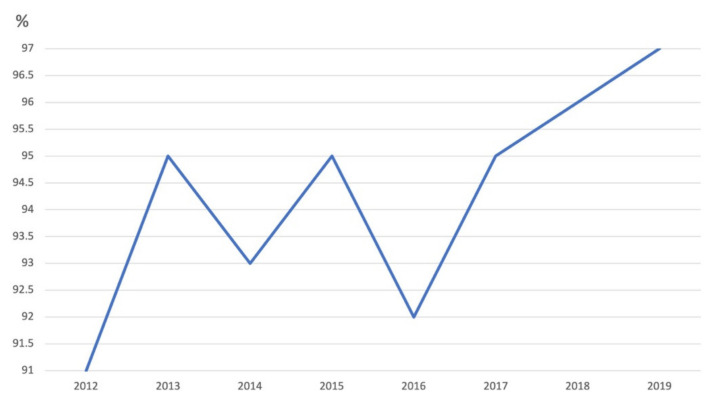
Diphtheria-pertussis-tetanus vaccine coverage (children ages 12–23 months) in Serbia, 2012–2019. Data: WHO.

**Figure 9:**
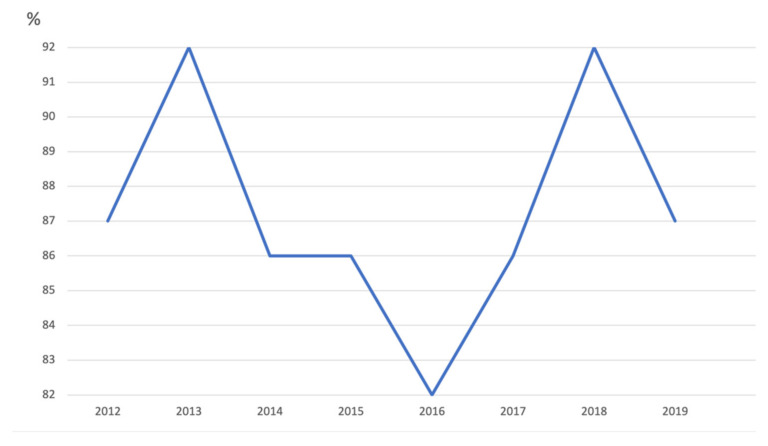
Measles vaccine coverage (children ages 12–23 months) in Serbia, 2012–2019. Data: WHO.

At the beginning of 2016, the National Assembly adopted a Law on Protection of the Population from Infectious Diseases. According to this Law, compulsory immunization is one of the measures for protection of the population from infectious diseases. It regulates fines for people who refuse immunization. Doctors are also subject to penalties if they oppose mandatory vaccination [38]. Three studies on Serbia analyze vaccine coverage, incidence, and seroprevalence, at regional and national levels. Patic et al. argue that MMR coverage in Vojvodina province was lower than 95% in 2012 (91%), 2014 (86%), 2015 (90%), 2016 (89%), and 2017 (78%). Coverage with the second dose of MMR increased significantly since 2015: 2015 (83.7%), 2016 (90.9%), 2017 (93.2%), thanks to the massive catch-up campaign for immunization against measles [30]. Ristic et al. argue that declining trend of measles incidence in Vojvodina in 1948–2017 was observed with wide variation in annual notification rates from 768.8/100,000 in 1970 to 0 during 12 different periods/years (2001–2006, 2008–2011, 2012, and 2016). Coverages for both MMR1 and MMR2 vaccines in Vojvodina increased from 78% and 93% (at the end of 2017) to 90% and 94% (during the first two months of 2018), respectively [31]. According to the surveillance report from the WHO vaccine coverage for all but MMR2 dropped by 2% to 5% in 2016 compared to 2015, with increase in 2017 to the level of 2015 and further increase in 2018 and 2019, by 2% for some vaccines in comparison to 2015 (DTP3, Hib3, Pol3). Figure 8 and Figure 9 show DTP and measles vaccine coverage in Serbia. Table 7 shows vaccination coverage and number of cases of corresponding diseases in Serbia in 2015–2019.

The incidence of measles dropped significantly in 2016 compared to 2015 (from 383 to 11) with a subsequent increase and topping in 2018 (5076) and only 22 in 2019. The incidence of mumps gradually decreased from 2016. The incidence of pertussis has increased from 2015 through 2018. The incidence of rubella decreased from 2016 [27]. Therefore, the data from these studies suggest that the 2016 law caused an immediate negative effect on the vaccine coverage with subsequent stabilization in the next years and even moderate increase in the coverage. This trend has been reflected in the incidence.

### 3.7. Ukraine

Since 2019, the provisions of article 15 of the Law on the Protection of the Population from Infectious Diseases stipulate that children who have not been vaccinated in accordance with the national immunization schedule are prohibited from attending educational institutions [39]. The two government reports analyze data on vaccine coverage and incidence. Table 8 shows vaccination coverage and number of cases of corresponding diseases in Ukraine in 2018–2020.

**Figure 10:**
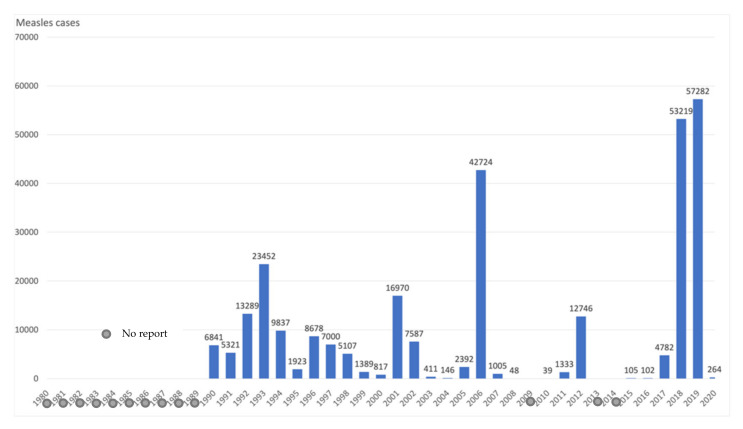
Number of reported measles cases in Ukraine, 1980–2020. Data: Public Health Center of the Ministry of Health of Ukraine; WHO.

**Figure 11:**
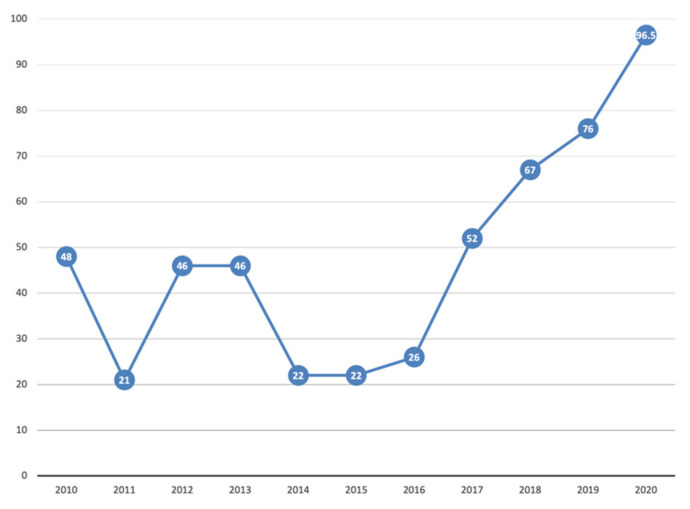
Vaccine coverage with three doses of the hepatitis B vaccine (one-year-old children) in Ukraine, 2010–2020. Data: Public Health Center of the Ministry of Health of Ukraine; WHO.

There has been a significant increase in vaccine coverage in 2020 compared to 2018. Polio vaccine coverage increased by 12 percentage points, DTP by 11 percentage points, HepB by 29 percentage points, and Hib by 25 percentage points. MMR1 increased by 2 percentage points in 2019 compared to 2018, but in 2020 it dropped by 10 percentage points to 83% [32]. In 2019 the incidence of diphtheria increased from 10 to 21 cases and the incidence of pertussis by 4.5%. The spread of measles slowed down in 2018, with an increase of 7.5% in 2019. In 2020, a dramatic reduction in the incidence of all the diseases covered by this review has been reported. The incidence of measles dropped from 135/100,000 to 0.63/100,000 [8]. The effect of COVID-19 preventive measures on the incidence of diseases covered by this review is evident. At the same time, COVID-19 did not cause major disruptions of routine vaccinations in Ukraine. Figure 10 and Figure 11 show the incidence of measles and HepB3 vaccine coverage in Ukraine.

The data show positive effect of the vaccination policy on vaccine coverage, in line with the incidence data and do not indicate any major negative effect neither on vaccine coverage nor on incidence.

### 3.8. Impact of COVID-19 Pandemic on Vaccination Coverage and Incidence of the Vaccine-Preventable Diseases

The impact of the COVID-19 pandemic should be considered while analyzing vaccine coverage and incidence data, especially in the countries that have recently introduced children vaccine mandates. Therefore, we obtained additional data relating to COVID-19 on the example of Germany, Ukraine, and France, from the studies included in the review. Regarding the incidence of the diseases covered by vaccination mandates, Germany and Ukraine demonstrate dramatic reduction in 2020. Matysiak-Klose et al. argue that a reduction of positive measles cases or reports can have various reasons: non-pharmacological measures (keeping your distance, wearing masks), schools and borders closures, and the decline in travel combined with a decline in cases across Europe have actually resulted in a decline in measles cases. On the other hand, measles cases could have been overlooked because measles sufferers did not seek medical help for fear of being infected with SARS-CoV-2 or the health authorities did not have time to allow the submission of reported cases of measles [14]. In 2020 and 2021, a significant reduction in the incidence of almost all the diseases covered by this review can be observed. In 2020 the incidence of HepB and mumps in Germany dropped by 25% and 43% respectively, compared to 2019. The reduction not only continued in 2021 but was in most cases even more significant than in 2020 [7,16]. Thus, in January–May 2021, only four cases of measles have been registered, compared to 75 cases during the same period in 2020. The incidence of pertussis in January–May 2021 dropped 10 times, compared to the same period in 2020. Ukraine follows this trend with reduction in the incidence of all the diseases covered by this review in 2020 compared to 2019, with the most significant being, measles by 215 times, mumps by 2.5 times, and rubella by 3.6 times [8]. In the case of Ukraine, COVID-19 did not cause any major disruption in the vaccinations according to children vaccination schedules. The vaccination coverage grew steadily in 2020 throughout 2019, except for MMR1, which dropped to 83.3% from 93% in 2019. The data confirm overall positive effect of COVID-19 preventive measures on incidence and no major negative effect on vaccination coverage in Ukraine [32]. At the same time, Taine at al. indicate that the pandemic negatively affected vaccination coverage in France, observed in the example of vaccines dispensation [13]. During the first 10 months of the COVID-19 pandemic in 2020 in France, all mandatory priming dose and booster dispensations were reduced as compared to the expected estimates based on the previous year. The reduction was particularly striking during the first 4 weeks of the first lockdown, especially for the MMR. During the immediate post-lockdown period, the counts for all mandatory vaccine dispensations remained slightly below what was expected.

## 4. Discussion

Our study explored the effect of mandatory childhood routine vaccination policies on vaccine coverage and incidence in Europe, as well as analyzed this effect in the context of the pandemic. Overall, the introduction of vaccine mandates in the countries included in the review did not show negative effects on vaccine coverage and incidence, except the immediate negative effect observed in Serbia with further improvement of the indicators. The mandates in Italy and France have affected positively the vaccine coverage and morbidity, with a high degree of certainty. Tightening vaccination policy in Latvia also showed positive effect, with a high degree of certainty on vaccination coverage and incidence, while the indicators in Moldova do not show a clear trend. A clear worsening trend has not been observed following the introduction of the mandate in Moldova. Drastic reduction in the incidence of vaccine-preventable diseases after the implementation of the mandates has been observed in Germany and Ukraine, mainly due to COVID-19 preventive measures. Despite the general reduction trend, the data on incidence may not give an accurate picture of the morbidity, because of possible underreporting of cases of diseases covered by this study during the pandemic. Vaccine coverage for almost all the vaccines has been significantly improved in Ukraine after the implementation of the mandate despite the COVID-19 pandemic.

The evidence of the positive effect is more solid from Italy and France, compared to other countries included in the review, due to a more systematic approach in introduction of mandatory vaccination, better surveillance, and availability of information. Fewer studies are available from Eastern Europe, and the transition from recommended to mandatory vaccination is not so distinct to determine the effect of the measures.

These findings are in line with the results of several other studies. Lee et al. analyzed the results of the implementation of mandates in the USA and Canada. The authors argue that introduction of mandatory vaccination for school entry and for middle school students has increased vaccine coverage both short-term and over time. At the same time, the researchers point at the necessity to approach the introduction of new mandates with caution, due to lack of evidence on the effectiveness of mandates in kindergartens, as well as limited information on mandates effectiveness in countries other than the US or recent introduction of mandates in the countries with no previous mandate [40]. Authors of another systematic review, based on studies from the USA and Australia, argue that there is no sufficient evidence to understand whether such interventions are effective, although mandates limiting access to education to vaccinated children may be effective for up to 6 years after the intervention [41]. Bechini at al. analyzed data from the WHO/UNICEF report to assess the impact of different vaccination policies on vaccination coverage in Europe and concluded that vaccine mandates are effective as a temporary solution [42]. Trentini et al. modeled and simulated enhancing vaccination coverage of routine programs and introducing mandatory vaccination at school entry on the residual measles susceptibility in the next 30 years in seven countries. The authors concluded that in order to maintain susceptible population below 7.5% up to 2050, the majority of countries (UK, Ireland, the USA, and Australia) need to increase vaccination coverage above 95% or introduce mandatory vaccination at school entry with the coverage above 40%. The researchers also point at the benefits of the introduction of mandatory vaccination for school enrollment in Italy and call for improving strategies targeting adults’ immunization. Furthermore, they argue that current vaccination policies are not sufficient to achieve and maintain measles elimination in most countries [43]. Vaz et al. analyzed the WHO and the European Centre for Disease Prevention and Control data on measles and pertussis and concluded that mandatory vaccination was associated with a 3.71 percentage point higher prevalence of measles vaccination and a 2.14 percentage point higher prevalence of pertussis vaccination in comparison with the countries that did not have mandatory vaccination. The authors did not find a significant association between mandatory vaccination and pertussis incidence. Vaz et el. found that mandatory vaccination and the magnitude of fines were associated with higher vaccination coverage and that mandatory vaccination was associated with lower measles incidence for countries with mandatory vaccination without nonmedical exemptions [44].

While the included studies on Italy, France, and the countries that most recently introduced vaccine mandates, such as Germany and Ukraine, do not allow to make a conclusion regarding the sustainability of the effect of the introduction of vaccine mandates, the examples of Latvia and Serbia demonstrate the stable positive effect of mandates on vaccine coverage and incidence in time.

Eastern European countries are often characterized by social, political, and historical-cultural distrust in the state. Studies explained that population of these countries place more trust in individuals (friends and family) and distrust the state authorities, seeing them more as enemies. Serbia is not an exception [45]. This might explain an immediate negative effect of introduction of sanctions for not vaccinating children there. An increase in concerns about vaccine safety in Serbia between 2015 and 2019 has also been found. The uneven trend in vaccines coverage and incidence of vaccine-preventable diseases in Moldova during the decade following the introduction of school enrollment vaccination mandate demonstrates ineffectiveness of application of such mandate in the context of maintaining the recommended vaccination policy. The population can resort to vaccination certificate forgery to comply with school enrollment requirements. Therefore, there is a lack of more systemic and unified approach to vaccination policy in the country.

The population in Eastern European countries has a higher level of concern about vaccine safety and effectiveness [46]. The trend continued during the COVID-19 pandemic. Gallup research on willingness to take the COVID-19 vaccine showed that only 43% on average said they would in Commonwealth of Independent States countries, and 46% would in non-EU-member countries in Europe [47]. Yet our analysis showed that, overall, the balance of benefits and harms of the effect of the introduction of tightening vaccination policies on vaccine coverage and incidence is in the favor of benefits, even in the case of Eastern Europe. Indeed, the introduction of recommended COVID-19 vaccination in Russia, the country that follows the above trend, did not lead to the desired coverage, which started growing after the introduction of mandatory COVID-19 vaccination of employees in a number of regions of Russia [48].

Considering the positive effect of the introduction of children vaccine mandates in Italy and France, these countries extrapolated this policy on COVID-19 vaccination. On 1 April 2021, Italy, the first country in Europe, adopted an emergency decree, according to which vaccination against COVID-19 became mandatory for health care workers. Those who refuse to get vaccinated can opt for transferring to duties with low risk of spreading the virus or to be suspended without pay for up to a year [49].

In July 2021 the French president, Emmanuel Macron, announced that vaccination would be mandatory for anyone who comes into contact with vulnerable people, including doctors, nurses, office staff, and volunteers. These professionals must be fully vaccinated against COVID-19 by 15 September or will risk not being paid [50].

The topic of children routine vaccination mandates is closely related to COVID-19 vaccination mandates, and it must be analyzed in the context of the COVID-19 pandemic, despite the difference between the diseases and the vaccines. The COVID-19 pandemic revealed the issues in vaccination policies that are especially evident in some of the countries. In many ways, the trends and patterns in attitudes to vaccines and in implementation of routine vaccination programs continued in the case of vaccinations against COVID-19. Countries where recommended vaccination policies have been effective, did not experience major issues achieving desired vaccine coverage with COVID-19 vaccines, like in the case of Spain [51]. Vaccination programs need periodical review and adaptation to changing situations. In case of a drop in vaccination coverage, increase of incidence or in case of a rapidly evolving situation like the COVID-19 pandemic, the evidence on vaccination program’s effectiveness is crucial.

This study presents new evidence on the effectiveness of mandatory vaccination by analyzing childhood routine vaccination mandates on the example of diverse European countries. Additionally, the study provides new evidence in the context of the COVID-19 pandemic. The search for information included 53 WHO member state countries. The evidence is based on 10 vaccine-preventable diseases. The review included gray literature, and the articles and reports are written in a variety of languages and obtained from a wide range of sources. This has allowed us to carry out more comprehensive search and increase the completeness of the evidence.

The limitation of this study is that it does not generate enough evidence on the medium- and long-term sustainability and effectiveness of the mandates. Data from a number of countries that have recently introduced vaccine mandates, were not available or limited. The impact of the COVID-19 pandemic on routine vaccination programs should be further analyzed. The analysis of evidence that includes other countries and regions is needed. It is necessary to better understand how to maintain the effectiveness of vaccination policies in the long run and how to combine mandates with other measures, such as raising awareness among the population.

## 5. Conclusions

The evidence collected and analyzed in this review allows us to conclude that the introduction of childhood vaccination mandates increases vaccine coverage and reduces the incidence of vaccine-preventable diseases when compared to the situation before the introduction of the mandates. The data from five countries provide high certainty of evidence and the effect of the policies varies between large, moderate, and small, depending on vaccine and country. The trends in the effects are similar in countries with comparable characteristics. Therefore, similar policies can be applied in similar countries, with predictable outcomes. Benefits of introduction of childhood routine vaccination mandates outweigh the harms in the countries struggling to achieve target vaccine coverage and reduce morbidity for vaccine-preventable diseases. The balance of benefits and harms will likely be different in the countries where recommended vaccination policies allow maintaining necessary coverage without experiencing major problems.

## Figures and Tables

**Table 1:** Characteristics of included studies.

Study	Country	Population	Study Period	Study Design	Vaccine/ Disease	Intervention	Outcomes	Results					
Cohen R. et al. (2020) [11]	France	1500 mothers of 0 to 17-month-old infants	2017–2019	Survey	Pertussis Hib HepB Measles- mumps- rubella (MMR)	Change from recommended to mandatory vaccination against 8 diseases for all children born from Jan 2018	VC	VC against pertussis and Hib was high and stable in 2019 (≥ 94%) compared to previous years. Among 250 children aged 6 to 8 months old, HepB VC, significantly increased from 88.7% in 2017 to 96.6% in 2019 (P < 0.01). Among 250 children aged 9 to 11-month-old, HepB VC significantly increased from 87.8% to 95.7% between 2017 and 2019 (P < 0.01). Among 250 children aged 15 to 17 months, HepB VC significantly increased between 2017 and 2019 (from 86.1% to 93.0%, P < 0.05). MMR VC increased from 85.5% in 2017 to 89.5% in 2019.					
Lévy-Bruhl D. et al. (2019) [12]	France	Children born in 2015–2018 in France	2016–2018	Registry analysis	Hexavalent HepB MMR	Change from recommended to mandatory vaccination against 8 diseases for all children born from Jan 2018	VC	Increase of VC against HepB from around 92% in 2017 to 98% in 2018. The increase in MMR first dose VC between 2017 and 2018 was 3.0%. This compared with a 0.3% gain in coverage between 2016 and 2017. The proportion of children under 1 year old, receiving a hexavalentvaccine increased from 93.1% in 2017 to 98.6% in 2018.					
Taine M. et al. (2021) [13]	France	Children ≤24 months old	2019–2020	Registry analysis	Penta/HexavalentMMR	Change from recommended to mandatory vaccination against 8 diseases for all children born from Jan 2018	VC, Vaccine dispensation	During the first 4 weeks of the first lockdown, the observed priming dose counts substantially decreased (RD: from −5.7% (95% CI −6.1; −5.2) for penta/hexavalent to −25.2% (95% CI −25.6; −24.8) for MMR), as did the booster counts (RD:−15.3% (95%CI −15.9; −14.7)) for penta/hexavalent. During 2020, MMR priming doses had the greatest shortfalls (n = 84,893). The number of booster dispensations between March 16 and December 20, 2020 had shortfalls reaching 21,140 doses of penta/hexavalent (RD −4.5% (95% CI −4.8; −4.2)).					
Matysiak-Klose D. et al. (2021) [14]	Germany	The population of Germany	2018–2021	Registry analysis	Rubella Measles	The law for protection against measles and to strengthen vaccination prevention in force since 1 March 2020.	Incidence	The number of reported rubella cases had consolidated at a low level in 2018 and 2019 (n = 58). In 2020: 18 rubella cases. In December 2020 Germany was granted an interruption of the endemic transmission for 36 months with retroactive effect for the years 2017 to 2019 and thus, the status of elimination of the rubella. 2018 (n = 545 measles cases) and 2019 (n = 515 measlescases). In 2020, 76 measles cases registered, decrease by 85.5% compared to previous years since 2016.					
Neugebauer M. et al. (2020) [15]	Germany	1594 adults	20 October 2019 to 24 April 2020	Survey	Measles	The law for protection against measles and to strengthen vaccination prevention in force since 1 March 2020.	Incidence, VC	19.3% of the participants were affected by the Measles Protection Act. Of these, only 77.5% had an immunity against measles, 14.0% wanted to be fully vaccinated against measles when the measles protection law came into force, whereby an immunity of 91.5% could be achieved. Assuming that participants with an unclear vaccination status or measles disease are immune, an immunity of > 95% is achieved. 86.4% of the children (2–17 years) had immune protection. The willingness of parents to have their children vaccinated because of the sanctions of the Measles Protection Act was only 0.8%.					
Robert Koch Institute (2021) [7]	Germany	The population of Germany	2019–2020	Registry analysis	Diphtheria Pertussis HepB Hib Measles Mumps Rubella Varicella	The law for protection against measles and to strengthen vaccination prevention in force since 1 March 2020.	Incidence	**Cases n**		**2019**		**2020**	
								Measles		515		75	
								Rubella		18		7	
								Diphtheria		15		16	
								Pertussis		10,315		3451	
								HepB		8941		6695	
								Hib		955		480	
								Mumps		593		338	
								Varicella		22,681		11,250	
Robert Koch Institute (2021) [16]	Germany	The population of Germany	2020–2021	Registry analysis	Diphtheria Pertussis HepB Hib Measles Mumps Rubella Varicella	The law for protection against Measles and to strengthen vaccination prevention in force since March 1, 2020.	Incidence	**Cases n**		**Jan–May** **2020**		**Jan–May** **2021**	
								Measles		75		4	
								Rubella		4		2	
								Diphtheria		9		0	
								Pertussis		2895		290	
								HepB		2942		3061	
								Hib		387		77	
								Mumps		265		38	
								Varicella		7300		2200	
Adamo G. et al. (2019) [17]	Italy	The population of Italy	2013–2018	Registry analysis	Measles Rubella	Extension of the number of mandatory vaccinations, for school attendance, from four to ten in July 2017.	Incidence, VC	In 2017, measles incidence increased to 88.4/106 population. In 2018, measles incidence was 43.3 /106 population. Rubella: incidence remained relatively low over the period considered. Congenital rubella syndrome: one case has been reported in Italy since 2014. Vaccination coverage for measles-containing vaccine increased by 5.7 percentage points in 2018 compared to 2016 with regard to the 1st dose (87.3% in 2016 vs 93.0% in 2018) and by 6.6 percentage points with regard to the 2nd dose (82.2% in 2016 vs 88.9% in 2018). Similar trend observed for the first and second doses of rubella-containing vaccine.					
Costantino C. et al. (2020) [18]	Italy	Children < 7 years old in Palermo	2016–2018	Registry analysis	Hexavalent Measles-mumps-rubella-varicella (MMRV)	Extension of the number of mandatory vaccinations, for school attendance, from four to ten in July 2017.	VC	Full cycle hexavalent coverage rates showed an increase of 1.4% and 2.5% at 24 and 36 months respectively, from 2016 to 2018. Moreover, in the same period, a 7.2% and a 10.5% increase of adherence to first dose of MMRV were observed at 24 and 36 months, respectively. Vaccination coverage rates for full vaccination cycle of MMRVin the Palermo Local Health Unit, among 6 years old children, showed an increase from 61% to 89.7% (+28.7%) from 2016 to 2018.					
Gianfedi V. et. al. (2019) [19]	Italy	Children < 24 months of age	2015–2017	Survey	Measles Polio	Extension of the number of Mandatory vaccinations, for school attendance, from four to ten in July 2017.	VC	Decrease in missed vaccination after the introduction of mandatory law for both polio and measles (−4.1%) with the consequent increase of VC. For measles vaccination the reason “found/contacted, but did not attend the appointment” was the most frequent (mean value 3.2%; 3.7% in 2015, 3.7% in 2016, and 2.3% in 2017), followed by “definitive informed dissent” (mean value 2.9%; 4.0% in 2015, 3.0% in 2016 and 1.8% in 2017).					
Gori D. et al. (2020) [20]	Italy	Children < 8 years old in Emilia- Romagna and Sicily regions	2009–2018	Registry analysis	MMR	Extension of the number of Mandatory vaccinations, for school attendance, from four to ten in July 2017.	VC	Observed 4.1% increase in Emilia-Romagna and 6.4% increase in Sicily in VC for MMR in 2017, and an additional 2.5% in Emilia-Romagna and 5.3% in Sicily in 2018. Both regions showed similar results; they achieved the lowest coverage rates in 2015 and showed an increase in the VC after the introduction of mandatory vaccinations. In 2018, both reached the starting point before the decrease.					
Signorelli C. et al. (2018) [21]	Italy	The population of Italy	2000–2017	Registry analysis	Polio Tetanus Diphtheria Pertussis HepB Hib Measles Mumps Rubella	Extension of the number of Mandatory vaccinations, for school attendance, from four to ten in July 2017.	VC	VC has increased since 2016 for both mandatory and recommended childhood vaccines. The increase has been consistent—although at different rates—in all regions and is highest for MMR, as compared to other vaccines.					
								VC		2016		2017	
								Polio		93.3		94.5	
								Tetanus		93.7		94.6	
								Diphtheria		93.6		94.6	
								Pertussis		93.6		94.6	
								HepB		93		94.3	
								Hib		93.1		94.2	
								Measles		87.3		91.7	
								Mumps		87.2		91.6	
								Rubella		87.2		91.6	
Signorelli C. et al. (2019) [22]	Italy	Children ≤ 24 months of age in Italy	2000–2018	Registry analysis	Poliomyelitis MMR	In 2007 Veneto region suspended mandatory immunization against polio, hepatitis B, tetanus, and diphtheria for children. Extension of the number of mandatory vaccinations, for school attendance, from four to ten in July 2017.	VC	Initially no significant drop in coverage rates in Veneto, as compared to previous years. Long-term data show for Veneto Region greater decreases in coverage rates (−5.0% for polio vaccine between 2006 and 2016), as compared to figures reported at the national level (−3.2% for polio vaccine in the same study period) and in other neighboring regions. In 2017 VC against polio was 94.5%, a 1.2% increase compared with 2016 with 11 regions exceeding 95%. MMR coverage was 91.6% for the year 2017, showing a 4.4% increase compared with 2016 (87.2%). The increasing trend in VC has continued in 2018. Data confirm a positive impact of the law on coverage rates which increased for MMR and polio by, respectively, 3.1% and 0.7% after 2017.					
Zanella B. et al. (2020) [23]	Italy	Pediatric and adolescent (1–18 years) residents of the province of Florence	December 2017– April 2018	Retrospective	Measles	Since 2017, the anti-measles Vaccination Became Compulsory in minors (0–16 years) for school attendance.	Seropreva-lence	No measles notification was reported. The overall seropositivity was 88.5%, and highest immunity level was found in the 5–9-year-old subjects (97.9%). Comparing these results with two serosurveys carried out in 2003 and 2005–2006, the current study highlighted a dramatic decrease in susceptibility towards measles (8.5%), with a lower value than in 2003 (30.8%) and in 2005–2006 (25.5%). The recent decrease in the susceptibility towards measles in the pediatric and adolescent population of Tuscany, together with the increased measles seroprevalence, is mainly due to all the preventive measures implemented in Italy.					
Heininger U. et al. (2016) [24]	Multi-country. Data on Latvia extracted	The population of Latvia	2000–2013	Registry analysis	Pertussis	Adoption in 2000 of “Vaccine Regulation” No. 330 which specifies that vaccination shall be mandatory for children against 10 vaccine-preventable diseases.	Incidence, VC	VC: From 1958 to 2004: 89.7–94.7% From 2005 to 2009: 92.3–98.1% Since 2010: 90.0–97.9% Approximately ten-fold decrease in the reported annual pertussis incidence rate between 2000 and 2011, before a dramatic increase in 2012. Over the period, the annual incidence of reported pertussis cases ranged from 0.4 to 13 cases per 100,000 population.					
Kantsone I. et al. (2016) [25]	Latvia	The population of Latvia	1994–2014	Registry analysis	Diphtheria	Adoption in 2000 of “Vaccine Regulation” No. 330 which specifies that vaccination shall be mandatory for children against 10 vaccine-preventable diseases.	Incidence, VC	Increasing incidence observed in 2000 (11.1/100,000). From 2000 to 2014, childhood VC with a third dose ranged from 91% to 98% and with a fifth dose from 92% to 98%. From 2000 to 2014, VC for adolescents (sixth dose at 15 years) ranged from 86% to 96%, decrease in coverage occurred from 96% in 2007 to 86% in 2014. Incidence increased from 0.1 per 100,000 inhabitants in 2010 to 0.7 in 2013. No cases in children were observed from 2009 to 2011, but new cases have emerged since 2012. Most cases occurred in adults.					
Wagner K. et al. (2012) [26]	Multi-country. Data on Latvia extracted	The population of Latvia	2000–2009	Registry analysis	Diphtheria	Adoption in 2000 of “Vaccine Regulation” No. 330 which specifies that vaccination shall be mandatory for children against 10 vaccine-preventable diseases.	Incidence, N of deaths	Diphtheria incidence decreased in Latvia from 111.22/million in 2000 to 2.67/million in 2009. During 2000–2009, a total of 32 deaths caused by diphtheria were reported in Latvia. 64 patients in Latvia recorded as fully vaccinated had classic respiratory diphtheria symptoms. Most of these patients were infected during a military outbreak in 2000 and would have been scheduled for primary vaccinations during the 1980s, when changes led to less intensive vaccination of children in the former Soviet Union.					
World Health Organization (2020) [27]	Latvia	The population of Latvia	1980–2019	Registry analysis	Diphtheria Tetanus Pertussis (DTP) HepB Hib Measles Mumps Rubella Polio Measles- containing vaccine (MCV)	Adoption in 2000 of “Vaccine Regulation” No. 330 which specifies that vaccination shall be mandatory for children against 10 vaccine-preventable diseases.	VC, incidence	**VC**	**2002**	**2001**	**2000**	**1999**	**1998**
								DTP1	99	100	100	93	99
								DTP3	97	97	96	95	94
								HepB3	98	96	95	94	9
								Hib3	87	84	79	76	47
								MCV1	98	98	97	97	97
								Pol3	98	97	96	95	94
								**Cases n**					
								Diphtheria	45	91	264	81	67
								Measles	-	1	0	0	3
								Mumps	231	6834	1949	41	-
								Pertussis	159	160	135	55	149
								Rubella	2578	358	62	91	-
								Tetanus	1	2	3		1
Bukova V. et al. (2017) [28]	Moldova	The population of Moldova	1993–2016	Registry analysis	Pertussis	From 2009 children are required to receive all vaccines in the national schedule to enroll in kindergartens and schools.	Incidence, VC	Reduction of incidence in 2009–2010 from 48 to 31 cases with increase in incidence in the following years. In 2009–2013 vaccination coverage was above 90%, and under 90% in the following years. The lowest incidence for the analyzed years was observed in 2005–2010—within 24 to 48 cases annually. Progressive reduction of the vaccine coverage observed in 2008–2015, from 95.4 to 89.7%, caused an increase in the incidence.					
Melnik A. et al. (2019) [29]	Moldova	The population of Moldova	2000–2018	Registry analysis	Measles	From 2009 children are required to receive all vaccines in the national schedule to enroll in kindergartens and schools.	Incidence, VC	In 2003–2017, the incidence dropped sharply, with a total of 181 cases of measles reported. No measles cases were reported in 2008–2011 and in 2015–2017. MMR1 VC: gradual decrease in 2003–2017, the lowest rate is observed in 2017—87.1%. In 2009–2012 VC was stable at the level of 90%. Moldova achieved the status of a country that eliminated measles in 2008–2017. Among the cases, children from one to ten years predominate (66.1%), cases among children under one year old (8.2%), adolescents (17.5%) and adults (8.2%).					
World Health Organization (2020) [27]	Moldova	The population of Moldova	1980–2019	Registry analysis	Diphtheria Tetanus Pertussis HepB Hib Measles Mumps Rubella Polio	From 2009 children are required to receive all vaccines in the national schedule to enroll in kindergartens and schools.	VC, incidence	**VC**	**2011**	**2010**	**2009**	**2008**	**2007**
								DTP1	96	93	95	96	95
								DTP3	93	90	94	90	92
								HepB3	96	98	95	97	95
								Hib3	78	63	94	-	-
								MCV1	91	97	91	95	96
								MVC2	97	98	98	97	95
								Pol3	96	97	95	96	93
								**Cases n**					
								Diphtheria	0	0	1	0	0
								Measles	0	0	0	0	10
								Mumps	143	144	292	29783	1757
								Pertussis	102	31	47	30	36
								Rubella	0	0	1	1	3
								Tetanus	0	2	0	0	0
Patić A. et al. (2020) [30]	Serbia	3404 participants from Vojvodina Province aged 0–84	May 2015– December 2017	Retrospective	Rubella	At the beginning of 2016, the National Assembly adopted a Law on Protection of the Population from Infectious Diseases, mandating vaccination.	Incidence, seroprevalence, VC	From 2008 to 2017, only three cases of rubella were reported (in 2009, in 2012, and in 2015). The highest number of seronegatives was in the youngest (1 year) age group (44.7%), followed by the group aged 24–49 (6.4%) and 2–11 years (6.2%). In the group of children aged 2–11 years, the level of anti-rubella IgG antibodies was higher since any of them have received both doses of the MMR vaccine. VC in Vojvodina was lower than 95% in 2012 (91%), 2014 (86%), 2015 (90%), 2016 (89%), and 2017 (78%). Coverage with the second dose of MMR increased significantly since 2015: 2015 (83,7%), 2016 (90,9%), 2017 (93,2%).					
Ristić M et al.(2019) [31]	Serbia	3199 residents of Vojvodina province aged 12 months and older	April 2015–June 2017	Retrospective	Measles	At the beginning of 2016, the National Assembly adopted a Law on Protection of the Population from Infectious Diseases, mandating vaccination.	Incidence, seroprevalence, VC	Declining trend of measles incidence in Vojvodina in 1948–2017 was observed with wide variation in annual notification rates from 768.8/100,000 in 1970 to 0 during 12 different periods/years (2001–2006, 2008–2011, 2012, and 2016). VC for both MMR1 and MMR2 vaccines in Vojvodina increased from 78% and 93% (at the end of 2017) to 90% and 94% (during the first two months of 2018), respectively. Findings showed that the main susceptible age group for measles transmission in the province are the subjects aged 20–39 years.					
World Health Organization (2020) [27]	Serbia	The population of Serbia	1980–2019	Registry analysis	Diphtheria Tetanus Pertussis HepB Hib Measles Mumps Rubella Polio	At the beginning of 2016, the National Assembly adopted a Law on Protection of the Population from Infectious Diseases, mandating vaccination.	VC, incidence	**VC**	**2019**	**2018**	**2017**	**2016**	**2015**
								DTP3	97	97	95	93	95
								HepB3	94	91	93	91	94
								Hib3	97	97	95	92	95
								MCV1	87	93	86	82	87
								MVC2	91	90	91	90	87
								Pol3	97	97	95	93	95
								**Cases n**					
								Measles	22	5076	721	11	383
								Mumps	-	13	37	40	41
								Pertussis	-	351	285	148	89
								Rubella	-	5	5	5	10
								Tetanus	-	0	2	4	4
Public Health Center of the Ministry of Health of Ukraine (2021) [32]	Ukraine	The population of Ukraine	2017–2020	Registry analysis	Polio DTP HepB Hib MMR	From 2019, unvaccinated children are prohibited from attending educational institutions.	VC	**VC**			**2020**	**2019**	**2018**
								DTP			80.1	80.5	69
								HepB			96.5	76	67
								Hib			83.6	80	58
								MMR1			83.3	93.2	91
								Polio			83	78.4	71
Public Health Center of the Ministry of Health of Ukraine (2021) [8]	Ukraine	The population of Ukraine	2018–2021	Registry analysis	Tetanus Diphtheria Pertussis Hepatitis B Hib Measles Mumps Rubella	From 2019, unvaccinated children are prohibited from attending educational institutions.	Incidence	**Cases/100,000**			**2020**	**2019**	**2018**
								Measles			0.63	135	125
								Mumps			0.39	0.90	0.92
								Pertussis			2.48	5.48	5.22
								Rubella			0.09	0.33	0.55
								HepB			1.74	3.11	3.42
								Cases n					
								Tetanus			12	15	18
								Diphtheria			0	21	10
								Hib			10	25	75

**Table 2:** Vaccination coverage in France, 2018–2020.

Vaccine	2018	2019	2020
Hexavalent, 3 doses at 21 months	84.1%	90.3%	90.5%
Hexavalent, including HepB among children vaccinated against diphtheria, tetanus, and polio, 1 dose at 8 months	96.4%	99.1%	99.4%
MMR, 1 dose at 12 months	90.8%	92.5%	92.2%
MMR, 2 doses at 33 months	81.6%	83.1%	84%

**Table 3:** Number of cases of measles, mumps, and rubella in Germany, 2019–2021.

Disease	January–May 2019	January–May 2020	January–May 2021
Measles N cases	422	75	4
Mumps N cases	210	265	38
Rubella N cases	10	4	2

**Table 4:** Vaccination coverage and number of cases of measles, mumps, and rubella in Italy, 2016–2019.

Vaccine/Disease	2016	2017	2018	2019
Measles VC, 1 dose at 24 months	87.3%	91.7%	93%	94.5%
Polio VC, 3 doses at 24 months	93.3%	94.5%	95.1%	95%
Measles N cases	862	5404	2682	1622
Mumps N cases	782	829	777	587
Rubella N cases	30	68	21	23

**Table 5:** Vaccination coverage and number of cases of corresponding diseases in Latvia, 1998–2002.

Vaccine	1998	1999	2000	2001	2002
DTP1	99%	93%	100%	100%	99%
DTP, 3 doses at 12 months	94%	95%	96%	97%	97%
HepB, 3 doses at 12 months	9%	94%	95%	96%	98%
Hib, 3 doses at 12 months	47%	76%	79%	84%	87%
Measles, 1 dose at 12 months	97%	97%	97%	98%	98%
Polio, 3 doses at 12 months	94%	95%	96%	97%	98%
Disease, N cases					
Diphtheria	67	81	264	91	45
Measles	3	0	0	1	-
Mumps	-	41	1949	6834	231
Pertussis	149	55	135	160	159
Rubella	-	91	62	358	2578
Tetanus	1		3	2	1

**Table 6:** Vaccination coverage and number of cases of corresponding diseases in Moldova, 2007–2011.

Vaccine	2007	2008	2009	2010	2011
DTP1	95	96	95	93	96
DTP, 3 doses at 12 months	92	90	94	90	93
HepB, 3 doses at 12 months	95	97	95	98	96
Hib, 3 doses at 12 months	-	-	94	63	78
Measles, 1 dose at 12 months	96	95	91	97	91
Measles, 2 doses at 7 years of age	95	97	98	98	97
Polio, 3 doses at 12 months	93	96	95	97	96
Disease, N cases					
Diphtheria	0	0	1	0	0
Measles	10	0	0	0	0
Mumps	1757	29,783	292	144	143
Pertussis	36	30	47	31	102
Rubella	3	1	1	0	0
Tetanus	0	0	0	2	0

**Table 7:** Vaccination coverage and number of cases of corresponding diseases in Serbia, 2015–2019.

Vaccine	2015	2016	2017	2018	2019
DTP, 3 doses at 12 months	95	93	95	97	97
HepB, 3 doses at 12 months	94	91	93	91	94
Hib, 3 doses at 12 months	95	92	95	97	97
Measles, 1 dose at 12 months	87	82	86	93	87
Measles, 2 doses at 7 years of age	87	90	91	90	91
Polio, 3 doses at 12 months	95	93	95	97	97
Disease, N cases					
Measles	383	11	721	5076	22
Mumps	41	40	37	13	-
Pertussis	89	148	285	351	-
Rubella	10	5	5	5	-
Tetanus	4	4	2	0	-

**Table 8:** Vaccination coverage and number of cases of corresponding diseases in Ukraine, 2018–2020.

Vaccine	2018	2019	2020
DTP, 3 doses at 12 months	69	80.5	80.1
HepB, 3 doses at 12 months	67	76	96.5
Hib, 3 doses at 12 months	58	80	83.6
Measles, 1 dose at 12 months	91	93.2	83.3
Polio, 3 doses at 12 months	71	78.4	83
Disease, N cases			
Diphtheria	10	21	0
Measles	53,219	57,282	264
Mumps	392	382	163
Pertussis	2214	2314	1041
Rubella	235	138	36
HepB	1449	1312	731
Tetanus	18	15	12
Hib	75	25	10

**Figure A1:**
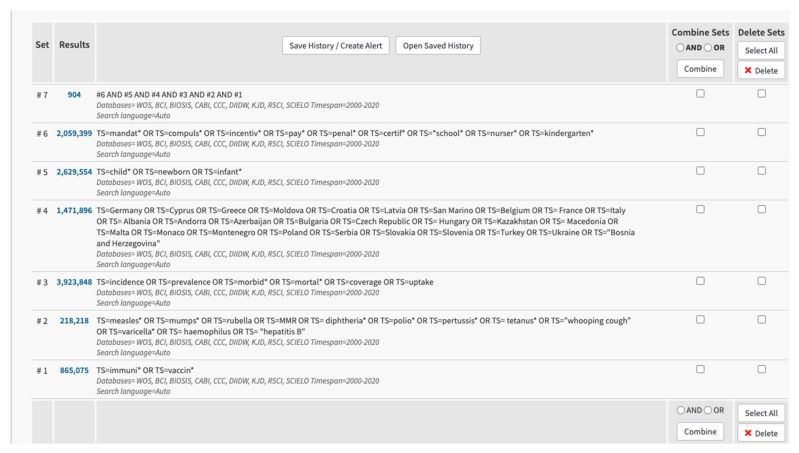
Example Search Strategy from Web of Science.

## Appendix A. Example Search Strategy from MEDLINE

Database: Ovid MEDLINE(R).

Search Strategy:

1. Immunization/(51045);
2. immuni*. mp. [mp = title, abstract, original title, name of substance word, subject heading word, floating sub-heading word, keyword heading word, organism supple-mentary concept word, protocol supplementary concept word, rare disease supplementary concept word, unique identifier, synonyms] (458065);
3. exp Vaccination/(86424);
4. vaccin*. mp. [mp = title, abstract, original title, name of substance word, subject heading word, floating sub-heading word, keyword heading word, organism supple-mentary concept word, protocol supplementary concept word, rare disease supplementary concept word, unique identifier, synonyms] (385553);
5. Immunization Programs/(11077);
6. 1 or 2 or 3 or 4 or 5 (708621);
7. exp Measles-Mumps-Rubella Vaccine/(2766);
8. exp Measles Vaccine/(8937);
9. exp Mumps Vaccine/(3788);
10. exp Poliovirus Vaccines/(7514);
11. exp Rubella Vaccine/(5147);
12. Vaccines, Combined/(2361);
13. Pertussis Vaccine/(5204);
14. Diphtheria-Tetanus Vaccine/(398);
15. Diphtheria-Tetanus-Pertussis Vaccine/(2897);
16. Chickenpox Vaccine/(2054);
17. Hepatitis B Vaccines/(9349);
18. Haemophilus Vaccines/(2991);
19. measles*. mp. [mp = title, abstract, original title, name of substance word, subject heading word, floating sub-heading word, keyword heading word, organism supple-mentary concept word, protocol supplementary concept word, rare disease supplementary concept word, unique identifier, synonyms] (28284);
20. mumps*. mp. [mp = title, abstract, original title, name of substance word, subject heading word, floating sub-heading word, keyword heading word, organism supple-mentary concept word, protocol supplementary concept word, rare disease supplementary concept word, unique identifier, synonyms] (11217);
21. rubella.mp. [mp = title, abstract, original title, name of substance word, subject heading word, floating sub-heading word, keyword heading word, organism supple-mentary concept word, protocol supplementary concept word, rare disease supplementary concept word, unique identifier, synonyms] (16142);
22. MMR.mp. [mp = title, abstract, original title, name of substance word, subject heading word, floating sub-heading word, keyword heading word, organism supplemen-tary concept word, protocol supplementary concept word, rare disease supplementary concept word, unique identifier, synonyms] (8295);
23. diphtheria*.mp. [mp = title, abstract, original title, name of substance word, subject heading word, floating sub-heading word, keyword heading word, organism sup-plementary concept word, protocol supplementary concept word, rare disease supplementary concept word, unique identifier, synonyms] (22281);
24. polio*.mp. [mp = title, abstract, original title, name of substance word, subject heading word, floating sub-heading word, keyword heading word, organism supplemen-tary concept word, protocol supplementary concept word, rare disease supplementary concept word, unique identifier, synonyms] (36359);
25. pertussis*.mp. [mp = title, abstract, original title, name of substance word, subject heading word, floating sub-heading word, keyword heading word, organism sup-plementary concept word, protocol supplementary concept word, rare disease supplementary concept word, unique identifier, synonyms] (30518);
26. tetanus*.mp. [mp = title, abstract, original title, name of substance word, subject heading word, floating sub-heading word, keyword heading word, organism supple-mentary concept word, protocol supplementary concept word, rare disease supplementary concept word, unique identifier, synonyms] (29199);
27. whooping cough.mp. [mp = title, abstract, original title, name of substance word, subject heading word, floating sub-heading word, keyword heading word, organism supplementary concept word, protocol supplementary concept word, rare disease supplementary concept word, unique identifier, synonyms] (9574);
28. varicella*.mp. [mp = title, abstract, original title, name of substance word, subject heading word, floating sub-heading word, keyword heading word, organism sup-plementary concept word, protocol supplementary concept word, rare disease supplementary concept word, unique identifier, synonyms] (14853);
29. haemophilus.mp. [mp = title, abstract, original title, name of substance word, subject heading word, floating sub-heading word, keyword heading word, organism supplementary concept word, protocol supplementary concept word, rare disease supplementary concept word, unique identifier, synonyms] (29227);
30. hepatitis B.mp. [mp = title, abstract, original title, name of substance word, subject heading word, floating sub-heading word, keyword heading word, organism sup-plementary concept word, protocol supplementary concept word, rare disease supplementary concept word, unique identifier, synonyms] (101028);
31. 7 or 8 or 9 or 10 or 11 or 12 or 13 or 14 or 15 or 16 or 17 or 18 or 19 or 20 or 21 or 22 or 23 or 24 or 25 or 26 or 27 or 28 or 29 or 30 (283610);
32. exp Epidemiology/(27044);
33. inciden*.mp. [mp = title, abstract, original title, name of substance word, subject heading word, floating sub-heading word, keyword heading word, organism supple-mentary concept word, protocol supplementary concept word, rare disease supplementary concept word, unique identifier, synonyms] (1010003);
34. prevalence.mp. [mp = title, abstract, original title, name of substance word, subject heading word, floating sub-heading word, keyword heading word, organism sup-plementary concept word, protocol supplementary concept word, rare disease supplementary concept word, unique identifier, synonyms] (729539);
35. Vaccination Coverage/(1296);
36. coverage.mp. [mp = title, abstract, original title, name of substance word, subject heading word, floating sub-heading word, keyword heading word, organism supple-mentary concept word, protocol supplementary concept word, rare disease supplementary concept word, unique identifier, synonyms] (132429);
37. uptake.mp. [mp = title, abstract, original title, name of substance word, subject heading word, floating sub-heading word, keyword heading word, organism supple-mentary concept word, protocol supplementary concept word, rare disease supplementary concept word, unique identifier, synonyms] (392943);
38. 32 or 33 or 34 or 35 or 36 or 37 (2162074);
39. Germany.mp. [mp = title, abstract, original title, name of substance word, subject heading word, floating sub-heading word, keyword heading word, organism sup-plementary concept word, protocol supplementary concept word, rare disease supplementary concept word, unique identifier, synonyms] (199711);
40. Cyprus.mp. [mp = title, abstract, original title, name of substance word, subject heading word, floating sub-heading word, keyword heading word, organism supple-mentary concept word, protocol supplementary concept word, rare disease supplementary concept word, unique identifier, synonyms] (2292);
41. Greece.mp. [mp = title, abstract, original title, name of substance word, subject heading word, floating sub-heading word, keyword heading word, organism supple-mentary concept word, protocol supplementary concept word, rare disease supplementary concept word, unique identifier, synonyms] (25388);
42. Moldova.mp. [mp = title, abstract, original title, name of substance word, subject heading word, floating sub-heading word, keyword heading word, organism supple-mentary concept word, protocol supplementary concept word, rare disease supplementary concept word, unique identifier, synonyms] (1042);
43. Croatia.mp. [mp = title, abstract, original title, name of substance word, subject heading word, floating sub-heading word, keyword heading word, organism supple-mentary concept word, protocol supplementary concept word, rare disease supplementary concept word, unique identifier, synonyms] (9361);
44. Latvia.mp. [mp = title, abstract, original title, name of substance word, subject heading word, floating sub-heading word, keyword heading word, organism supple-mentary concept word, protocol supplementary concept word, rare disease supplementary concept word, unique identifier, synonyms] (2035);
45. San Marino.mp. [mp = title, abstract, original title, name of substance word, subject heading word, floating sub-heading word, keyword heading word, organism sup-plementary concept word, protocol supplementary concept word, rare disease supplementary concept word, unique identifier, synonyms] (115);
46. Belgium.mp. [mp = title, abstract, original title, name of substance word, subject heading word, floating sub-heading word, keyword heading word, organism supple-mentary concept word, protocol supplementary concept word, rare disease supplementary concept word, unique identifier, synonyms] (27470);
47. France.mp. [mp = title, abstract, original title, name of substance word, subject heading word, floating sub-heading word, keyword heading word, organism supple-mentary concept word, protocol supplementary concept word, rare disease supplementary concept word, unique identifier, synonyms] (129353);
48. Italy.mp. [mp = title, abstract, original title, name of substance word, subject heading word, floating sub-heading word, keyword heading word, organism supplemen-tary concept word, protocol supplementary concept word, rare disease supplementary concept word, unique identifier, synonyms] (124903);
49. Albania.mp. [mp = title, abstract, original title, name of substance word, subject heading word, floating sub-heading word, keyword heading word, organism supple-mentary concept word, protocol supplementary concept word, rare disease supplementary concept word, unique identifier, synonyms] (1394);
50. Andorra.mp. [mp = title, abstract, original title, name of substance word, subject heading word, floating sub-heading word, keyword heading word, organism supple-mentary concept word, protocol supplementary concept word, rare disease supplementary concept word, unique identifier, synonyms] (71);
51. Azerbaijan.mp. [mp = title, abstract, original title, name of substance word, subject heading word, floating sub-heading word, keyword heading word, organism sup-plementary concept word, protocol supplementary concept word, rare disease supplementary concept word, unique identifier, synonyms] (2015);
52. Bulgaria.mp. [mp = title, abstract, original title, name of substance word, subject heading word, floating sub-heading word, keyword heading word, organism supple-mentary concept word, protocol supplementary concept word, rare disease supplementary concept word, unique identifier, synonyms] (8195);
53. Czech Republic.mp. [mp = title, abstract, original title, name of substance word, subject heading word, floating sub-heading word, keyword heading word, organism supplementary concept word, protocol supplementary concept word, rare disease supplementary concept word, unique identifier, synonyms] (11565);
54. Hungary.mp. [mp = title, abstract, original title, name of substance word, subject heading word, floating sub-heading word, keyword heading word, organism supple-mentary concept word, protocol supplementary concept word, rare disease supplementary concept word, unique identifier, synonyms] (23035);
55. Kazakhstan.mp. [mp = title, abstract, original title, name of substance word, subject heading word, floating sub-heading word, keyword heading word, organism sup-plementary concept word, protocol supplementary concept word, rare disease supplementary concept word, unique identifier, synonyms] (3718);
56. Macedonia.mp. [mp = title, abstract, original title, name of substance word, subject heading word, floating sub-heading word, keyword heading word, organism sup-plementary concept word, protocol supplementary concept word, rare disease supplementary concept word, unique identifier, synonyms] (1391);
57. Malta.mp. [mp = title, abstract, original title, name of substance word, subject heading word, floating sub-heading word, keyword heading word, organism supplemen-tary concept word, protocol supplementary concept word, rare disease supplementary concept word, unique identifier, synonyms] (1609);
58. Monaco.mp. [mp = title, abstract, original title, name of substance word, subject heading word, floating sub-heading word, keyword heading word, organism supple-mentary concept word, protocol supplementary concept word, rare disease supplementary concept word, unique identifier, synonyms] (415);
59. Montenegro.mp. [mp = title, abstract, original title, name of substance word, subject heading word, floating sub-heading word, keyword heading word, organism sup-plementary concept word, protocol supplementary concept word, rare disease supplementary concept word, unique identifier, synonyms] (887);
60. Poland.mp. [mp = title, abstract, original title, name of substance word, subject heading word, floating sub-heading word, keyword heading word, organism supple-mentary concept word, protocol supplementary concept word, rare disease supplementary concept word, unique identifier, synonyms] (57784);
61. Serbia.mp. [mp = title, abstract, original title, name of substance word, subject heading word, floating sub-heading word, keyword heading word, organism supple-mentary concept word, protocol supplementary concept word, rare disease supplementary concept word, unique identifier, synonyms] (5550);
62. Slovakia.mp. [mp = title, abstract, original title, name of substance word, subject heading word, floating sub-heading word, keyword heading word, organism supple-mentary concept word, protocol supplementary concept word, rare disease supplementary concept word, unique identifier, synonyms] (4886);
63. Slovenia.mp. [mp = title, abstract, original title, name of substance word, subject heading word, floating sub-heading word, keyword heading word, organism supple-mentary concept word, protocol supplementary concept word, rare disease supplementary concept word, unique identifier, synonyms] (4551);
64. Turkey.mp. [mp = title, abstract, original title, name of substance word, subject heading word, floating sub-heading word, keyword heading word, organism supple-mentary concept word, protocol supplementary concept word, rare disease supplementary concept word, unique identifier, synonyms] (54574);
65. Ukraine.mp. [mp = title, abstract, original title, name of substance word, subject heading word, floating sub-heading word, keyword heading word, organism supple-mentary concept word, protocol supplementary concept word, rare disease supplementary concept word, unique identifier, synonyms] (17769);
66. Bosnia and Herzegovina. mp. [mp = title, abstract, original title, name of substance word, subject heading word, floating sub-heading word, keyword heading word, organism supplementary concept word, protocol supplementary concept word, rare disease supplementary concept word, unique identifier, synonyms] (2868);
67. 39 or 40 or 41 or 42 or 43 or 44 or 45 or 46 or 47 or 48 or 49 or 50 or 51 or 52 or 53 or 54 or 55 or 56 or 57 or 58 or 59 or 60 or 61 or 62 or 63 or 64 or 65 or 66 (679377);
68. 6 and 31 and 38 and 67 (2312);
69. limit 68 to “all child (0 to 18 years)” (1521);
70. limit 69 to year = “2000 -Current” (1174).
